# Mutations in *LAMB2* Are Associated With Albuminuria and Optic Nerve Hypoplasia With Hypopituitarism

**DOI:** 10.1210/clinem/dgz216

**Published:** 2019-11-26

**Authors:** Mona Tahoun, Jennifer C Chandler, Emma Ashton, Scott Haston, Athia Hannan, Ji Soo Kim, Felipe D’Arco, D Bockenhauer, G Anderson, Meei-Hua Lin, Salah Marzouk, Marwa H Saied, Jeffrey H Miner, Mehul T Dattani, Aoife M Waters

**Affiliations:** 1 Clinical and Chemical Pathology Department, Faculty of Medicine, Alexandria University, Egypt; 2 UCL Great Ormond Street Institute of Child Health, University College London, UK; 3 Great Ormond Street Hospital NHS Foundation Trust, London, UK; 4 Division of Nephrology, Department of Medicine, Washington University School of Medicine, St Louis, Missouri

**Keywords:** *LAMB2*, Pierson syndrome, optic nerve hypoplasia syndrome

## Abstract

**Context:**

Mutations in *LAMB2*, encoding the basement membrane protein, laminin β2, are associated with an autosomal recessive disorder characterized by congenital nephrotic syndrome, ocular abnormalities, and neurodevelopmental delay (Pierson syndrome).

**Case description:**

This report describes a 12-year-old boy with short stature, visual impairment, and developmental delay who presented with macroscopic hematuria and albuminuria. He had isolated growth hormone deficiency, optic nerve hypoplasia, and a small anterior pituitary with corpus callosum dysgenesis on his cranial magnetic resonance imaging, thereby supporting a diagnosis of optic nerve hypoplasia syndrome. Renal histopathology revealed focal segmental glomerulosclerosis. Using next-generation sequencing on a targeted gene panel for steroid-resistant nephrotic syndrome, compound heterozygous missense mutations were identified in *LAMB2* (c.737G>A p.Arg246Gln, c.3982G>C p.Gly1328Arg). Immunohistochemical analysis revealed reduced glomerular laminin β2 expression compared to control kidney and a thin basement membrane on electron microscopy. Laminin β2 is expressed during pituitary development and *Lamb2*^*–/–*^ mice exhibit stunted growth, abnormal neural retinae, and here we show, abnormal parenchyma of the anterior pituitary gland.

**Conclusion:**

We propose that patients with genetically undefined optic nerve hypoplasia syndrome should be screened for albuminuria and, if present, screened for mutations in *LAMB2*.

Optic nerve hypoplasia syndrome (ONH) (also referred to as septo-optic dysplasia, SOD) represents a clinical spectrum associated with visual, pituitary, and severe central nervous system structural abnormalities (1, 2). Traditionally, the cardinal features of the controversial term, *SOD*, involved 2 or more of the following: (1) pituitary hypoplasia with isolated or combined pituitary hormone deficiencies, (2) ONH, and/or (3) midline brain defects. Risk factors include maternal exposure to recreational or prescription drugs, alcohol, diabetes, and viral infection. Mutations in genes encoding transcription factors that regulate eye, forebrain, and pituitary development have been identified in a small proportion of patients with ONH with hypopituitarism (1). HESX1 homeobox 1 (*HESX1*) encodes a homeobox protein involved in forebrain and early pituitary development in mice. Mutations in *HESX1* account for less than 1% of hypopituitarism ± ONH. An increasing number of genes implicated in ONH have been reported and include other transcription factors important for eye and forebrain development (*OTX2*, *SOX2*, *PAX6*, *NR2F1*, *VAX1*, and *ATOH7*), whereas others identified regulate cellular processes such as RNA splicing, chromatin remodeling, and the microtubular network (1).

Compound heterozygous mutations in *LAMB2*, encoding laminin β2, an extracellular matrix glycoprotein, were herein identified in a patient with 3 cardinal features of an ONH associated with growth hormone (GH) deficiency phenotype who also had proteinuric kidney disease. Screening for albuminuria may lead to further identification of *LAMB2* mutations in genetically undefined ONH diagnoses.

## Methods

Ethics approval was obtained from the Great Ormond Street Hospital NHS Foundation Trust. Following informed consent for genetic testing, targeted next-generation sequencing of *ACTN4*, *ADCK4*, *CD2AP*, *COQ2*, *COQ6*, *INF2*, *ITGA3*, *LAMB2*, *LMX1B*, *MYO1E*, *NPHS1*, *NPHS2*, *PDSS2*, *PLCE1*, *PTPRO*, *SMARCAL1*, *TRPC6*, and *WT1* was undertaken for nephrotic syndrome and *HESX1*. Patient and control kidney sections were stained with anti–laminin β2 (1:500, AMAb91096, Atlas) and anti-Podocin antibodies (1:100, P0372, Sigma) as previously described (3). Pituitary tissue of *Lamb2*^*–/–*^ and *Lamb2*^*+/+*^ mice (4) were stained with hematoxylin and eosin (H&E) and 3,3’-diaminobenzidine (DAB) as previously described (3).

## Case History

**Figure 1.**
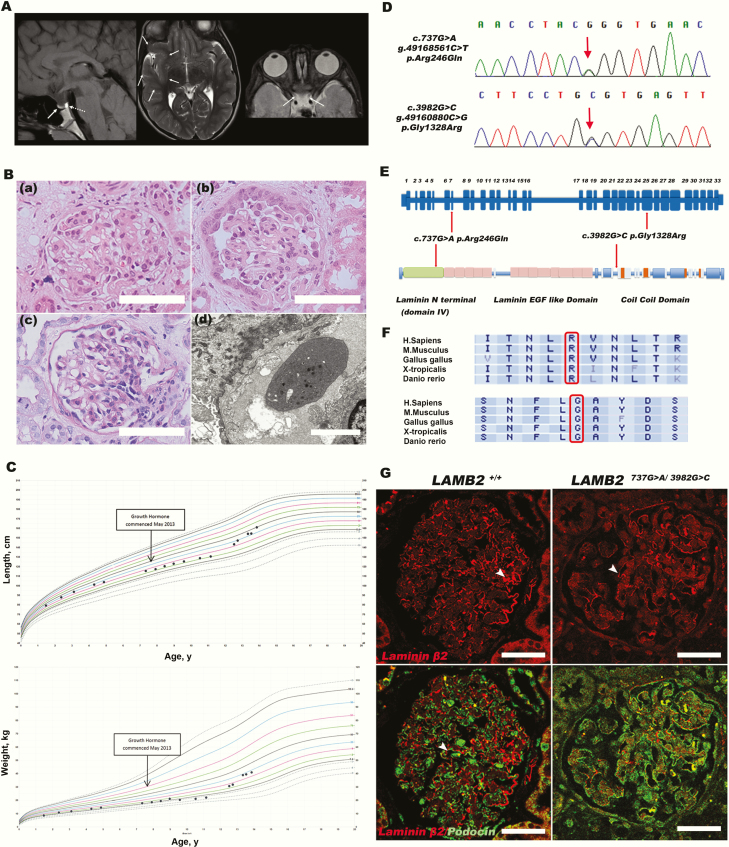
A, Sagittal T1-weighted imaging (WI) of the pituitary region shows a small adenohypophysis (WI) (arrow in A), with a normal T1 posterior hyperintense focus corresponding to the neurohypophysis (dotted arrow in A). Note the reduction of the bulk of the posterior aspect of the corpus callosum. Axial T2-WI shows a dysplastic cortex in the right anterior temporal and insular regions (arrows in B). Axial T2-WI of the orbits shows small optic nerves (arrows in C) and bilateral buphthalmos. B, Hematoxylin and eosin (H&E) and periodic acid–Schiff (PAS) staining of patient renal biopsy sections: a and b, H&E shows segmental sclerosis, narrowing of the Bowman space, and adhesions to its capsule; c, PAS staining shows mesangial proliferation with diffuse sclerosis and obliteration of the tip of the capillary tuft; d, Electron microscopy reveals a thin, lamellated glomerular basement membrane (GBM) and podocyte foot process effacement; scale bar 50 μm. C, Growth chart showing height (cm) and weight (kg) of the proband from ages 1 to 14 years; initiation of growth hormone (arrow). D, Sanger sequencing confirmed compound heterozygous *LAMB2* mutations: c.737G>A, Chr3: g.49168561C>T, p.Arg246Gln and c.3982G>C, Chr3:g.49160880C>G, and p.Gly1328Arg. E, Location of *LAMB2*-detected variants at the level of exons and protein. The c.737G>A variant is located in exon 7 encoding part of the laminin N-terminal (LN′) domain, whereas the c.3982G>C variant is located in exon 25 encoding part of the laminin coiled–coil domain (LCC′) of laminin β2 protein. The LN′ domain is responsible for laminin trimer polymerization to form the basement membrane. The LCC′ domain is involved in the assembly of individual laminin α, β, and γ chains into trimers. F, Conservation of the *LAMB2* amino acids that are altered in the patient: c.737G>A and c.3982G>C (upper and lower rows, respectively) alongside disparately related species. G, Dual immunofluorescence staining of laminin β2 in a patient renal biopsy (*LAMB2* 737G>A/ 3983G>C; mutant) compared to time zero protocol renal transplant biopsy (*LAMB2*^+/+^; control). The figure shows reduced expression of laminin β2 (red) in the mutant, seen in a reduced thickness and integrity and/or continuity of the GBM compared to the control.

A male infant presented at age 4 months with roving eye movements and impaired visual function. Born at 35 weeks’ gestation to a healthy mother age 33 years, he weighed 1.93 kg (–1.47 SD score [SDS]) and measured 42 cm (–2.13 SDS). No exposure to recreational drugs, alcohol, or prescription medications was reported, and his parents were nonconsanguineous. By age 31 months, he had global developmental delay with marked hypotonia. Cranial magnetic resonance imaging revealed bilateral hypoplastic intraorbital optic nerves and anterior pituitary hypoplasia with a global reduction of white matter. The bulk of the posterior corpus callosum was reduced (Fig. 1A). The septum pellucidum was present.

At age 5 years, he presented with recurrent macroscopic hematuria associated with hypoalbuminemia (25 g/L) and significant albuminuria (urine albumin to creatinine ratio was 1329 mg/mmol) with normal renal function. A renal biopsy showed focal segmental glomerulosclerosis (FSGS) and a thin, lamellated glomerular basement membrane (GBM) with podocyte foot process effacement (Fig. 1B).

On follow-up, the patient had short stature: His height was 117.7 cm (–1.4 SDS) with a weight of 18.5 kg (–2.54 SDS) at the chronological age of 7.9 years (Fig. 1C). Investigations revealed a low insulin-like growth factor 1 (IGF1) of 29 ng/mL and normal insulin-like growth factor–binding protein 3 (2.48 ng/L), normal thyroxine and thyrotropin, with a low free 3,5,3′-triiodothyronine of 5.3 pmol/L (6.2-9.5 pmol/L). A glucagon stimulation test revealed a peak GH concentration of 4.5 μg/L, suggesting GH insufficiency. At the start of the test, the basal glucose was 2.8 mmol/L with a nadir of 2.3 mmol/L, reflecting significant hypoglycemia. The peak cortisol to glucagon stimulation testing was normal, measuring 819 nmol/L. Measured prolactin concentrations ranged between 80 and 244 mU/L (normal: 44-479 mU/L). At a chronological age of 10.5 years, the patient’s bone age was delayed at 9 years, and therefore the low IGF1 could not be solely accounted for by undernutrition. Given the features of GH insufficiency along with his poor height velocity at 3.7 cm per year with a low IGF1 and neuroradiological findings, daily subcutaneous GH injections at 10 IU/m^2^/week were commenced (Fig. 1C). Following commencement of GH, his growth rate initially improved to 6.6 cm per year from a growth rate of 3.7 cm per year before commencement of GH. We suspect that adherence to GH treatment may have been an issue initially. Following transfer of the patient’s care to his grandparents, the excellent growth rate observed from age 12 years occurred while he was still prepubertal. At age 12.74 years, his height velocity was 10.6 cm per year, with pubertal ratings of G2 P1 A1 and testicular volumes of 2 mL on the right and 3 mL on the left. His bone age at that stage was 10.4 years.

Genetic testing for nephrotic syndrome revealed compound heterozygous missense mutations in *LAMB2*, the gene encoding laminin β2, a structural component of the GBM, previously implicated in Pierson syndrome (OMIM 609049) (5) (Fig. 1D). One mutation in exon 7 (c.737G>A, p.Arg246Gln) was originally reported (5), and the other, identified in exon 25 (c.3982 G>C, p.Gly1328Arg), was novel. Both variants were predicted to be deleterious to protein function using PolyPhen and SIFT software analyses (Fig. 1E) and affected highly conserved amino acids (Fig. 1F). No mutations were detected in *HESX1*, and the clinical phenotype did not match that documented for *SOX2* or *OTX2* mutations. Reduced glomerular expression of laminin β2 was observed in the patient biopsy compared to the control (Fig. 1G).

**Figure 2.**
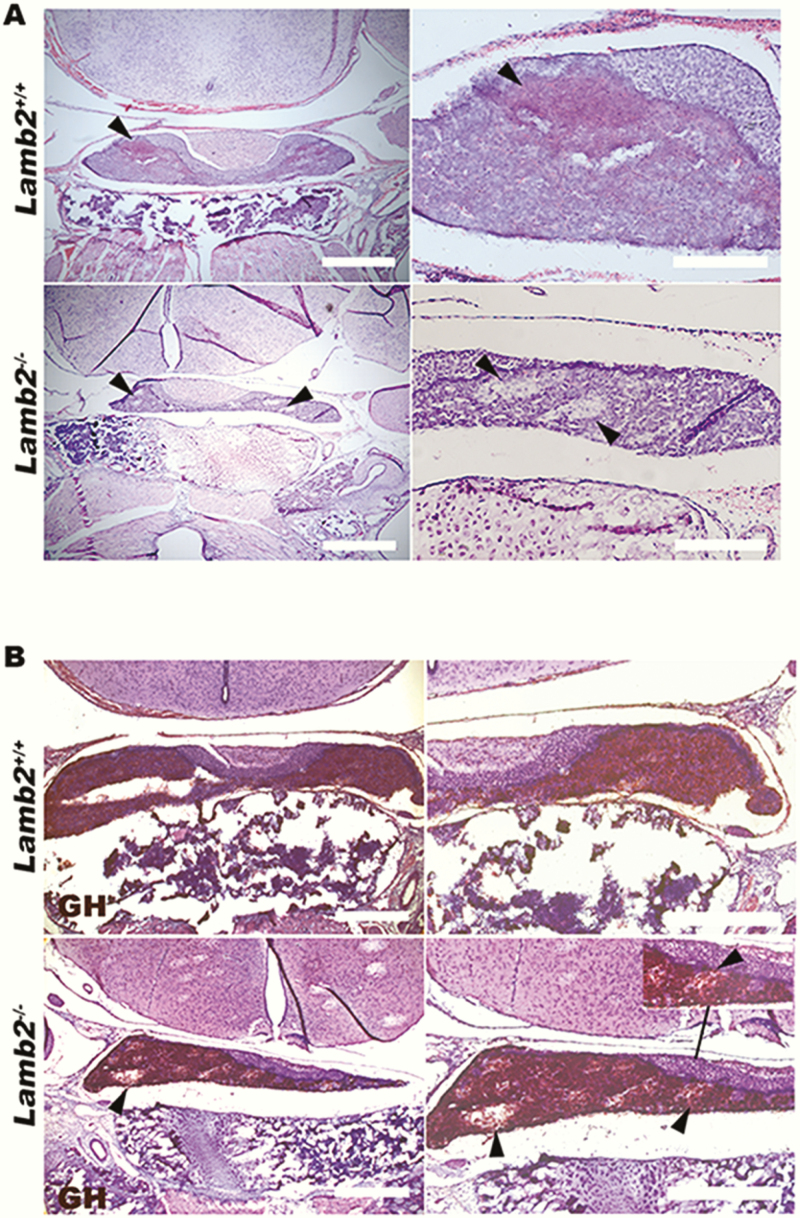
Hematoxylin and eosin (H&E) and 3,3’-diaminobenzidine (DAB) immunostaining of pituitary gland sections from wild-type and knockout *Lamb2* mice. A, The upper row shows H&E staining of anterior pituitary sections from *Lamb2*^*+/+*^ mice, which show uniform staining across the gland. The lower row shows anterior pituitary sections from the *Lamb2*^*–/–*^ mice, which show evidence of abnormal cell staining, seen as randomly distributed patches of unstained cells (indicated with arrow heads), imaged at magnifications of 5× (left) and 20× (right); scale bar of 500 μm and 200 μm, respectively. B, Growth hormone (GH) DAB immunostaining confirmed the abnormality of these cell clusters in *Lamb2*^*–/–*^ pituitary sections, which show negative staining for GH (lower row; indicated with arrow heads) compared to *Lamb2*^*+/+*^ mice (upper row); scale bar of 500 μm.

H&E staining of pituitary sections of *Lamb2*^*–/–*^ mice suggested abnormal morphology of the anterior pituitary parenchyma, exhibited by cellular clusters that were not evident in their wild-type littermates (Fig. 2A). Somatropin signal was absent in *Lamb2*^*–/–*^ compared to *Lamb2*^*+/+*^ pituitaries (Fig. 2B). Because the patient had isolated GH deficiency, analysis of other pituitary cell types was not undertaken.

## Discussion

Following presentation with recurrent macroscopic hematuria and albuminuria, compound heterozygous mutations in *LAMB2*, encoding laminin β2, were identified in a boy with short stature, visual impairment, and developmental delay. Investigations revealed ONH associated with anterior pituitary hypoplasia and GH deficiency. Although the septum pellucidum was present, dysgenesis of the posterior corpus callosum was evident, thereby supporting the traditional diagnosis of ONH with GH deficiency and midline defects. Several publications have highlighted that ONH with hypopituitarism now represents a spectrum of developmental defects involving the eye, neural retina, and forebrain (including the pituitary) with a range of midline defects that do not always involve an absent septum pellucidum.

Albuminuria associated with FSGS has not yet been reported in the ONH spectrum. Interestingly, nonsense and truncating mutations in *LAMB2* are associated with Pierson syndrome, an autosomal recessive disorder characterized by congenital nephrotic syndrome, ocular abnormalities (commonly microcoria), muscular hypotonia, and neurological deficits (5, 6). Indeed, Pierson syndrome may be within the ONH spectrum. Hypomorphic missense mutations have been reported with milder phenotypes, manifesting later in childhood, whereby defective secretion of the mutant laminin β2–trimer leads to compromised GBM integrity (7). The other variant (p.G1328R) is a novel missense mutation, in which glycine is replaced by an arginine at amino acid 1328 in the coiled coil domain of the peptide. Supporting evidence for a pathogenic mutant laminin β2 protein was the finding of a thin GBM on electron microscopy and reduced glomerular laminin β2 expression on patient kidney sections.

To date, genes implicated in ONH play a role in the transcriptional regulation of pituitary and forebrain development (1). In situ hybridization studies have revealed expression of laminin isoforms throughout pituitary morphogenesis (8, 9). In early murine gestation, laminin β2 messenger RNA is expressed in the epithelium of the Rathke pouch and by mid-gestation within the pars distalis and tuberalis (9). Expression later extends to the parenchyma and marginal cell layers of the anterior and intermediate pituitary lobes as well as the vasculature of the anterior lobe, both in late gestation and the early postnatal period (9). Laminin β2 expression is observed within the parenchyma and vasculature of the anterior lobe in the adult pituitary (9).

Our examination of *Lamb2*^*–/–*^ mice revealed abnormal parenchymal morphology of the anterior pituitary compared to controls, and *Lamb2*^*–/–*^ mice exhibit stunted growth (4, 10). Further investigations involving assessment of pituitary function with measurement of GH levels will be of interest. In *Lamb2*^*–/–*^ mice the outer segment of the rod photoreceptor layer is associated with disorganized synapses in the outer plexiform layer and a reduced physiological response (4, 10). Human and murine findings both support the proposal that mutations in *LAMB2* may underlie genetically undefined ONH, and urine dipstick testing may alert the clinician to screen for mutations in *LAMB2*.

## Abbreviations

DAB: 3,3’-diaminobenzidine
HESX1: homeobox expressed in embryonic stem cells 1
GBM: glomerular basement membrane
LAMB2: laminin β2
SOD: septo-optic dysplasia
SOX2: SRY (sex-determining region Y)-related HMG box 2
OTX2: orthodenticle homeobox 2

## Additional Information

***Disclosure Summary:*** The authors have nothing to disclose.

***Data Availability:*** All data generated or analyzed during this study are included in this published article or in the data repositories listed in References.
